# Mechanochemical synthesis of hyper-crosslinked polymers: influences on their pore structure and adsorption behaviour for organic vapors

**DOI:** 10.3762/bjoc.15.112

**Published:** 2019-05-24

**Authors:** Sven Grätz, Sebastian Zink, Hanna Kraffczyk, Marcus Rose, Lars Borchardt

**Affiliations:** 1 Anorganische Chemie, Ruhr-Universität Bochum, Universitätsstraße 150, 44801 Bochum, Germany; 2 Institute of Inorganic Chemistry, Technische Universität Dresden, Dresden, Germany; 3 Fachgebiet Technische Chemie II, Technische Universität Darmstadt, Darmstadt, Germany

**Keywords:** hyper-crosslinked polymers, mechanochemistry, microporous, solvent-free, vapor sorption

## Abstract

This study elucidates a mechanochemical polymerization reaction towards a hyper-crosslinked polymer as an alternative to conventional solvent-based procedures. The swift and solvent-free Friedel–Crafts alkylation reaction yields a porous polymer with surface areas of up to 1720 m^2^g^−1^ and pore volumes of up to 1.55 cm^3^g^−1^. The application of LAG (liquid-assisted grinding) revealed a profound impact of the liquid´s boiling point on the textural properties of the obtained polymer materials. Finally, the materials are characterized by vapour sorption experiments with benzene and cyclohexane.

## Introduction

The widespread use of microporous materials in areas like gas storage, gas separation, and catalysis has led to the development of a wide variety of these materials [1]. While inorganic materials such as activated carbons, porous metal oxides, and zeolites have been investigated for decades, hybrid materials such as metal-organic frameworks [2], purely organic frameworks such as crystalline covalent-organic frameworks [3], and amorphous POPs (porous organic polymers) [4] are currently in the focus of research. The modular bottom-up building concept of these organic materials allows for tailoring of materials properties towards desired applications. POPs synthesis can be achieved by a huge variety of reactions ranging from Friedel-Crafts alkylations [5], cross-coupling reactions [6] and cyclotrimerizations [7], to amine-based chemistry with Schiff base reactions [8], imidisation, and amidisation reactions [9]. In the recent past, a strong focus was also set on the development of HCPs (hyper-crosslinked polymers) [10]. Typically, they are formed by a non-directed aromatic substitution (Friedel–Crafts alkylation) either by intramolecular functional groups or using external crosslinkers. These reactions yield very high crosslinking degrees in amorphous framework structures and hence, enable highest specific BET (Brunauer–Emmett–Teller) surface areas of up to 2000 m^2^g^−1^ for fully amorphous materials [10]. Typically, chloromethyl or methoxy groups are used to crosslink aromatic building blocks by using stoichiometric or even excess amounts of FeCl_3_ as catalyst. Recently, also a metal-free Brønsted acid-catalyzed reaction using trifluoromethanesulfonic acid or sulfuric acid was developed yielding basically the same polymer but avoiding residual traces of the metal [11]. These synthetic pathways often afford a gel from the dissolved monomers upon the crosslinking that is subsequently washed and dried with a significant volume loss. Still, a high amount of permanent micro- and mesoporosity is retained. This gel formation is kinetically fast and thus, hard to control. Hence, an initial intensive mixing is crucial to achieve a homogeneous material. Also, due to the shrinkage upon the drying step, a high flexibility of the framework remains as an intrinsic property. Thus, the pore volume and specific surface area that is permanently accessible can be increased by a severe swelling behaviour especially during the adsorption and combined with absorption of organic vapors or liquids. The swelling is fully reversible, thus, rendering these materials interesting adsorbents with a dynamic adsorption behaviour.

However, the low solubility of POPs is a main challenge in their synthesis protocols. Solution-based procedures, for example, suffer from almost instant precipitation and thus only produce materials with a low degree of polymerization [10]. In the recent past it has been shown that for reactions, where the solubility of the reactants or products is an issue, mechanochemistry – a field which is currently gaining momentum – can be a promising workaround [12–20]. It has already been established as a versatile tool for the synthesis of several porous materials [21–29] and polymers [30–42].

**Figure 1:**
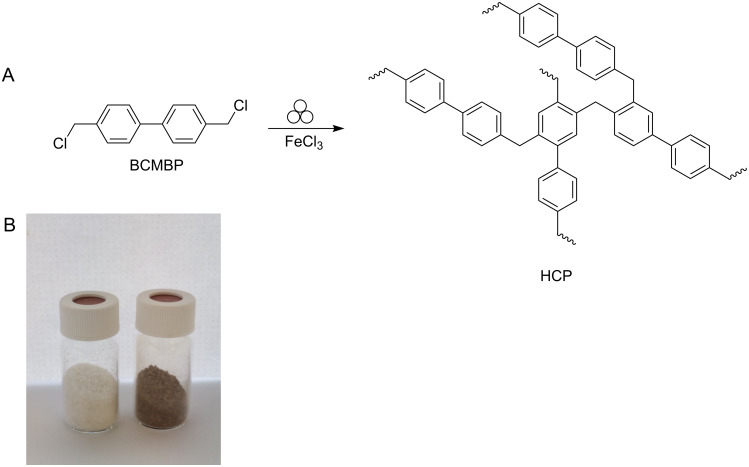
A: Mechanochemical polymerization of BCMBP (4,4’-bis(chloromethyl)-1,1’-biphenyl) towards the porous polymer HCP. B: Picture of a vial of BCMBP (left) and LAG-HCP (right).

In this contribution, we employed mechanochemistry for a protocol for the solvent-free crosslinking of HCP (Figure 1). This is supported by an investigation of chemical and milling parameters on the yield and surface area of the resulting polymer.

## Results and Discussion

### Polymer synthesis and characterization

In our standard procedure HCP was synthesised via a mechanochemical reaction. This was accomplished by transferring BCMCP (4,4’-bis(chloromethyl)-1,1’-biphenyl) and six equiv of anhydrous iron(III) chloride as mediator into a 45 mL zirconium oxide milling vessel filled with 22 balls (10 mm, 3.19 ± 0.05 g) of the same material (Figure 1). Consequently, the vessel was transferred into a (Pulverisette 7) premium line (Fritsch GmbH) and milled at 500 rpm for 35 minutes. After the synthesis, the resulting polymers were washed with 200 mL of water and 100 mL of ethanol and dried at 80 °C for 12 hours to yield a beige powder (denoted NG-HCP (neat grinding-HCP)). For more details on the experimental procedure see Supporting Information File 1.

**Figure 2:**
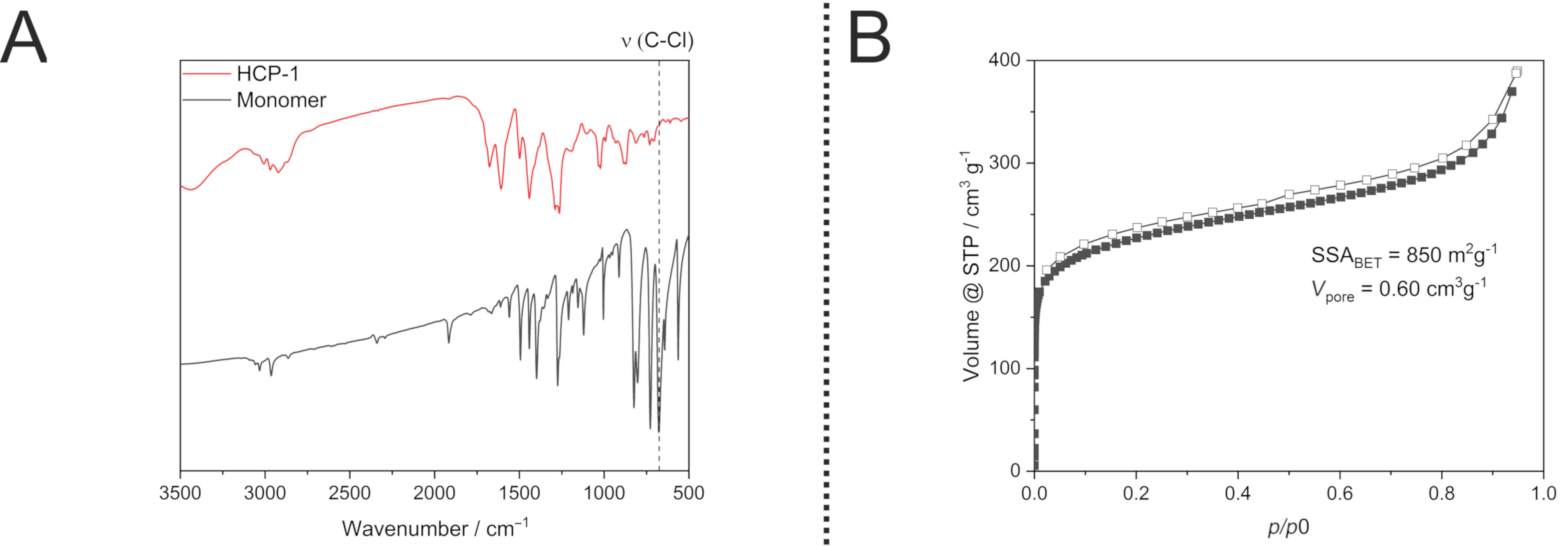
A: IR spectra of the monomer BCMBP and NG-HCP showing a decrease of the C–Cl vibration after the reaction. B: Low pressure nitrogen isotherm of NG-HCP demonstrating the textural properties.

After validating the complete insolubility in common organic solvents (like DCM, ethanol, DMF, chlorofrom, *n*-hexane), the nature of the polymer was investigated via IR spectroscopy. In the past the C–Cl vibration at 680 cm^−1^ has commonly been used to establish whether the desired crosslinking via the chloromethylene groups has occurred [43]. While this vibration is dominant in the monomer, it has completely vanished in the IR (infrared) spectrum of the polymer (Figure 2A). Moreover, several other changes in the spectrum are observed for the polymer as well. Especially in the region of the C–H_oop_ the vibration is shifted to 885 cm^−1^ hinting towards one isolated hydrogen atom which further confirms the cross-linking between the monomers. The creation of these aliphatic connections is also evident based on the appearance of vibrations around 1400 cm^−1^ [43]. As reported in the literature for the solvent-based protocol the polymer obtained through this mechanochemical route is also amorphous as visualized by the XRD (X-ray diffraction) patters recorded (Supporting Information File 1, Figure S1) [43].

Furthermore, NG-HCP similarly shows a high thermal stability with a decomposition onset in air (Supporting Information File 1, Figure S2) at around 310 °C. The residual mass of only 1.9% confirms the absence of both, excessive abrasion and oxidant inside the polymer. This is further supported by the SEM-EDX (scattering electron microscope, energy-dispersive X-ray spectroscopy) measurements showing both Cl and Fe contents below the limit of detection (Supporting Information File 1, Figure S3). Scattering electron microscopy also revealed an agglomerated morphology of the samples (Supporting Information File 1, Figure S4).

After successfully establishing the occurrence of the cross-linking reaction and investigating the physical and thermal properties of the polymer, the textural properties of the NG-HCP were evaluated utilizing nitrogen physisorption. While the first tries directly yielded a microporous polymer (SSA_BET_ = 850 m^2^g^−1^, *V*_p_ = 0.60 cm^−3^g^−1^, Figure 2B in Supporting Information File 1), the SSA (specific surface area) achieved with the classical solvent-based approach (Sol-HCP, SSA_BET_ = 1450 m^2^g^−1^, *V*_p_ = 1.55 cm^−3^g^−1^) [11] could not be reached. In order to improve this property, we varied the mechanochemical reaction parameters such as milling time and amount of oxidant in a systematic (DOE) design of experiments approach (see Supporting Information File 1 for details). While changes in these parameters translated to variations in the specific surface, it was not possible to achieve surface areas as high as the solution-based method solely by adapting the aforementioned parameters. The most influential parameter was determined to be the rotational speed of the ball mill. At 200 rpm this led to a low SSA (7–66 m^2^g^−1^) due to an incomplete polymerization, while 800 rpm resulted in a medium SSA (600–820 m^2^g^−1^). This observation is hinting towards a partial degradation of the porosity by the high energy input and the resulting thermal and frictional stress of the material and reinforces our prior findings regarding the synthesis of porous polymers inside a ball mill [30,35].

### Development of vessel pressure during the reaction

**Figure 3:**
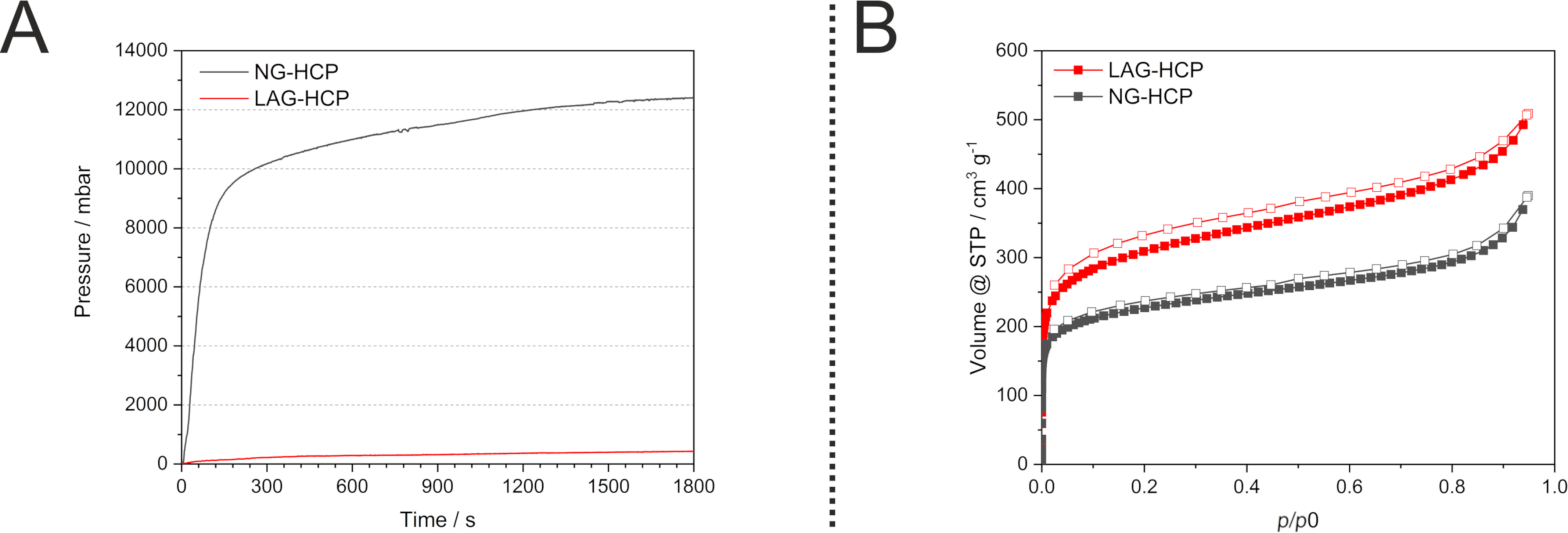
A: Evolution of pressure in the course of the reaction measured by the GTM system. The addition of 1 mL of Et_2_O is sufficient to drastically reduce the released pressure. B: Comparison of the low pressure nitrogen isotherm of NG-HCP and LAG-HCP.

In an attempt to track and understand the kinetics of the mechanochemical polymerisation further, we employed the so-called GTM-system (gas pressure and temperature measurement system), which allows us to measure the temperature and pressure inside the milling vessel during the milling process. While a release of gas was observed when opening the vessel, the GTM measurement revealed that even small amounts of the monomer (3.2 mmol) lead to a swift and rise in vessel pressure exceeding 12 bar (Figure 3A) caused by the released HCl in the course of the reaction. This behaviour can be handled under laboratory conditions but is hampering the potential scale up of the process. In the recent past we have already observed this behaviour for other reactions including iron(III) chloride [42,44], and thus we have proposed several countermeasures which greatly reduce the vessel pressure during such reactions. For this polymerization reaction we decided to add small quantities of liquid to dissolve the gas, as the method of choice. At first 1 mL of ethanol was added to the reaction mixture prior to the milling. This measure resulted in a decrease of vessel pressure of the whole course of the reaction. With diethyl ether instead of ethanol the pressure could be reduced even more limiting the maximum pressure to 0.42 bar (Figure 3B).

### Influence of the LAG additive

**Figure 4:**
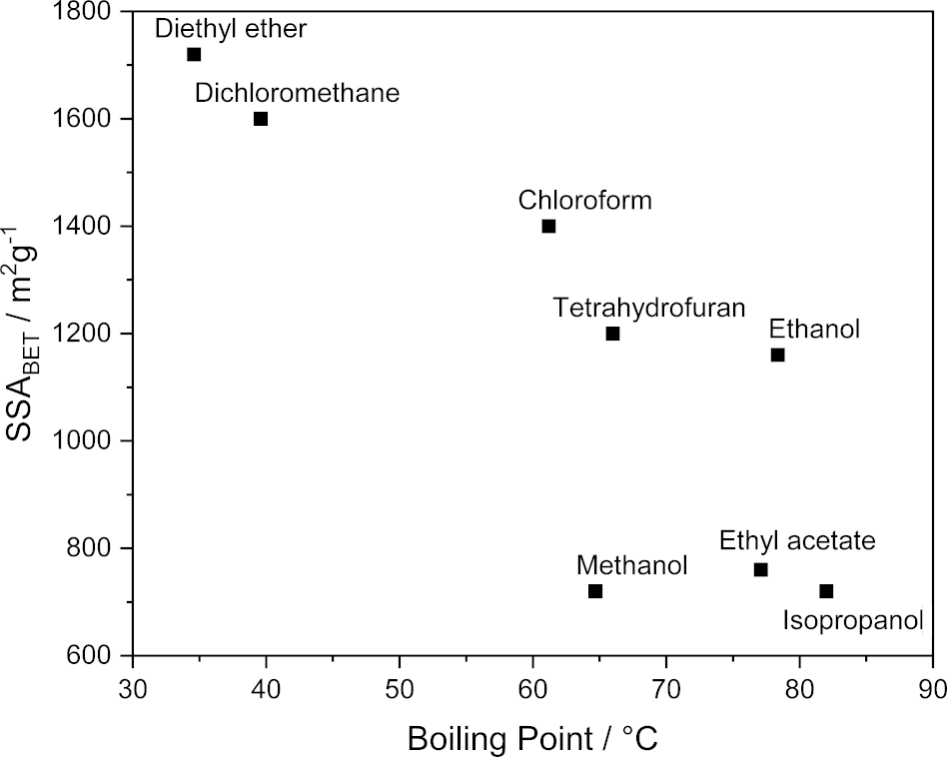
The correlation between the liquids’ boiling point and the SSA of the polymer. In general, a lower boiling point of the LAG additive leads to a higher SSA.

Interestingly, besides decreasing the vessel pressure, this 1 mL of solvent also affected the textural properties of the polymer positively. Consequently, we went on to investigate the influence of the LAG reagent by changing the nature of the latter. The surface area of the polymer was increased up to 1720 m^2^g^−1^ when utilizing diethyl ether (Table 1, entry 7), therefore almost doubled, while the pore volume is also growing. In the pore size distribution one can observe a shift from 0.75 nm micropores for NG-HCP to a mixture of 0.67 and 0.97 nm micropores for LAG-HCP (Figures S5 and S6 in Supporting Information File 1). In addition, the amount of mesopores in the sample is also slightly higher for the LAG sample. The solubility of the released gas inside the liquid seems to play a minor role since good results can be obtained with either dichloromethane (poor HCl solvent) as well as diethyl ether (good HCl solvent). On the other hand, the boiling point of the added liquid seems to be an important parameter for the surface area of the synthesised polymer, with lower boiling point solvents resulting in higher surface areas than their high boiling point counterparts (Figure 4). This might indicate that the additive acts as a porogene in the synthesis of the polymer, with the vapour being a more effective than the liquid. However, to test this hypothesis a profound control over the vessel temperature is necessary which cannot be achieved with our current experimental setup but is generally possible [45]. Nevertheless, the GTM systems showed that macroscopic temperatures exceeding the boiling points of dichloromethane and diethyl ether have been reached in the course of the experiments. Since the best results were obtained for diethyl ether (Table 1, entry 7) this sample has been denoted LAG-HCP and was investigated further.

**Table 1:** Influence of the LAG additive on the textural properties of HCP^a^.

Entry	Liquid additive	SSA_BET_^b^ [m^2^g^−1^]	*V*_Pore_^c^ [cm^3^g^−1^]

1	–	850	0.60
2	ethanol	1160	0.98
3	methanol	720	0.61
4	chloroform	1400	0.91
5	dichloromethane	1550	1.12
6	isopropanol	720	0.52
7^d^	diethyl ether	1720	1.06
8	ethyl acetate	760	0.51
9	tetrahydrofuran	1200	1.00
10^e^	–	1450	1.55

^a^All samples were synthesised under the following conditions: 35 min, 500 rpm, 6 equiv FeCl_3_, 22 balls á 10 mm, 1 mL of liquid additive; ^b^according to the Brunauer–Emmett–Teller theory, utilizing the Rouquerol method; ^c^determined at *p*/*p*_0_ = 0.99; ^d^in the following denoted as LAG-HCP; ^e^solution-based references, Sol-HCP.

### Vapor physisorption

Since HCP is highly hydrophobic (proven before by water vapor physisorption [11]), the adsorption of non-polar adsorptives in the gas and liquid phase is strongly favored. Recently, the advantage of HCP over various commonly available adsorbents in the selective liquid phase adsorption of least polar component in aqueous solution has been demonstrated [46–47]. Herein, the adsorption properties for the organic vapors benzene and cyclohexane are reported and the differences between LAG- HCP and the solvent-based reference material Sol-HCP are demonstrated.

**Figure 5:**
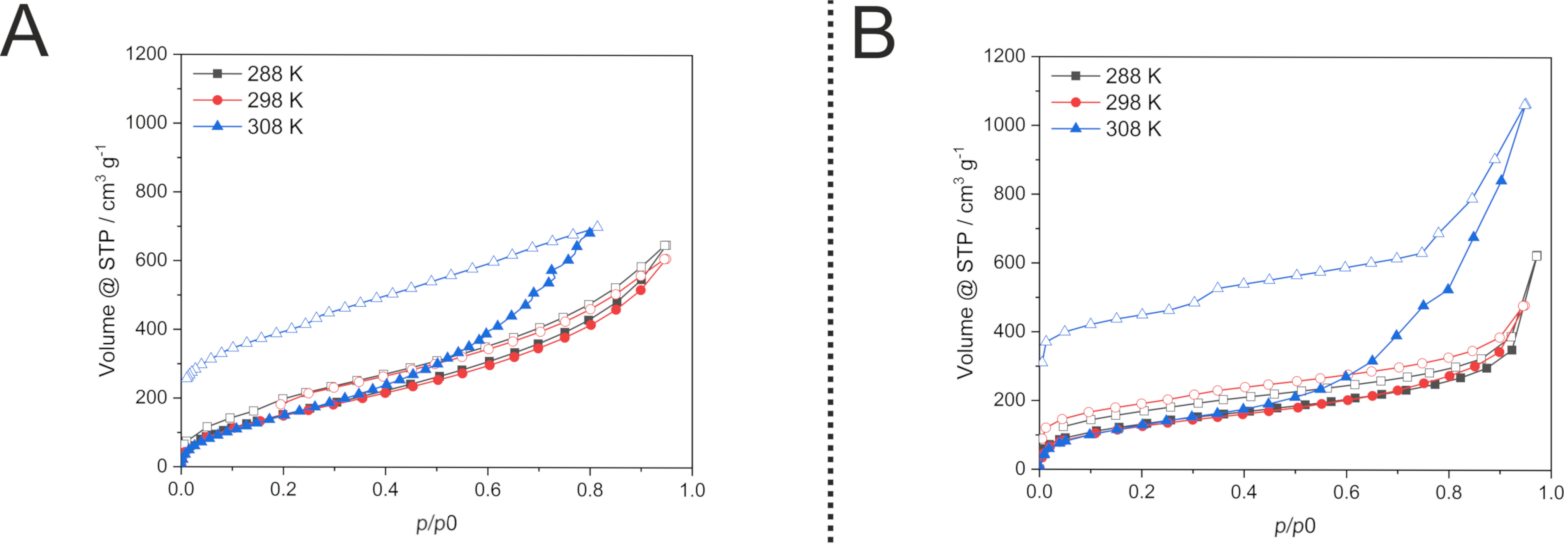
Physisorption isotherms of benzene vapour at different temperatures on HCP synthesised classically from solution (left, A) and mechanochemically (right, B). Filled symbols denote adsorption and open symbols desorption, respectively.

First of all, is has to be noted that Sol-HCP (reference according to Schute et al. [11]) showed a specific BET surface area of 1450 m^2^g^−1^, hence, slightly below the LAG-HCP with a SSA of 1720 m^2^g^−1^. Still, it showed a higher total pore volume of 1.55 cm^3^g^−1^ due to the higher ratio of mesopores and hence, a lower share of micropore volume (0.24 cm^3^g^−1^). Also, the pore size distribution proved to be rather broad (as reported before) compared to the mechanochemically synthesised material. For both materials the overall uptake of benzene is higher than for previously reported HCP materials [48], resulting from the large BET surface areas (Figure 5A).

Compared to nitrogen physisorption, the benzene vaporsorption isotherms show a much more pronounced hysteresis over the whole relative pressure range. This effect can be explained by the strong interactions of the benzene molecules with the aromatic surface of the polymer via π–π interactions. The rather strong adsorption at the inner surface is overlapped by pore filling due to a condensation-like effect. Thereby, swelling results in a reversible structural change. Interestingly, this swelling effect occurs only moderately up to a temperature of 298 K. With further increasing temperature the swelling is much more pronounced with a significantly higher uptake at high relative pressure by condensation and a very pronounced hysteresis upon pressure-driven desorption (Figure 5B). A very similar behaviour is observed for both HCP samples LAG-HCP and Sol-HCP. However, the Sol-HCP shows a steeper slope of the adsorption isotherm due to the broader pore size distribution. In contrast, the LAG-HCP with the narrow PSD shows a more pronounced uptake at low relative pressures.

**Figure 6:**
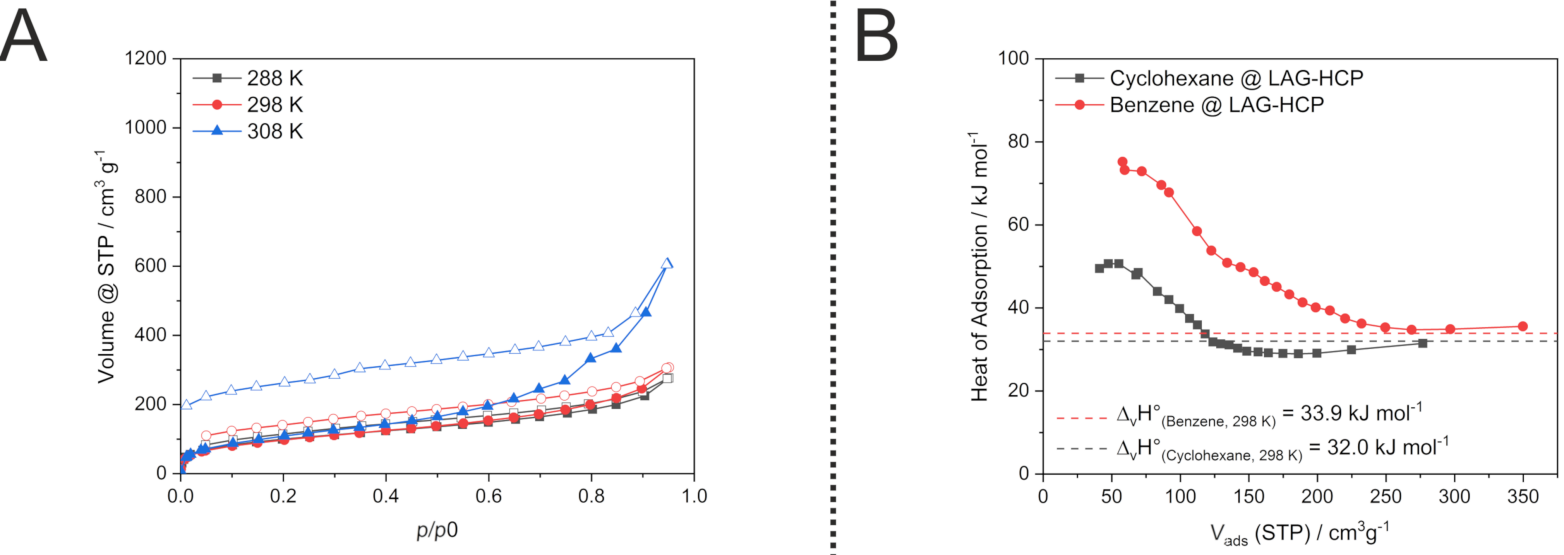
Physisorption isotherms of cyclohexane on mechanochemically synthesised HCP at temperatures 288, 298 and 303 K (left) and isosteric heat of adsorption of benzene and cyclohexane on HCP synthesised mechanochemically (LAG-HCP) (right).

In addition, cyclohexane vapour was measured in the same temperature range (Figure 6A). Over the whole relative pressure region a lower uptake is observed compared to the benzene vapour. In a similar fashion, but slightly weaker pronounced, an adsorption-desorption-hysteresis is found at higher temperature >298 K due to severe swelling. The difference in the uptake towards a favoured adsorption of benzene already points towards the potential for an adsorptive separation process of aromatic and aliphatic molecules with very similar physical properties.

To quantify the strength of adsorptive interactions from isotherms at various temperatures the isostatic heat of adsorption is calculated. Although in case of flexible materials with varying textural properties during adsorption and hence, a varying surface potential, this method should only be applied with great care and only used for a relative comparison of similar materials [49]. Absolute values calculated by this method are prone to great errors and should not be over-interpreted. Still, in our case the mechanochemically synthesised HCP shows an adsorption enthalpy at low loading of benzene of 70–80 kJ mol^−1^ that steadily decreases until it approximates the condensation enthalpy of pure benzene (Figure 6B). Interestingly, the heat of adsorption of cyclohexane is much lower with a maximum at low loading of approximately 50 kJ mol^−1^ and also decreasing until the condensation enthalpy is reached. Also, the condensation enthalpy of cyclohexane is reached at ca. 40% lower loading compared to benzene. This data points out the significantly favoured adsorption of benzene due to aromatic π–π interaction in comparison to the significantly weaker bonding of aliphatic cyclohexane.

## Conclusion

Summing up, we have demonstrated that mechanochemistry can be a suitable alternative in the synthesis of hyper-crosslinked polymers. By avoiding chlorinated solvents (typically 1,2-dichloroethane), the synthesis of this promising material can be undertaken in a greener, faster, and cheaper fashion. The obtained materials show surface areas as high as 1720 m^2^g^−1^ with a narrower pore size distribution compared to solvent-based analogues. It has been found that the addition of small amounts of liquid (LAG) is not only reducing the vessel pressure during the synthesis, but is also beneficial towards the textural properties of the material. In organic vaporsorption experiments the adsorption of benzene was favoured over cyclohexane by strong π–π interaction with the aromatic framework. This has been proven by differences in uptake as well as by comparison of isosteric heat of adsorptions as direct indicator of the adsorption interactions.

## Experimental

In our standard procedure HCP was synthesised through a mechanochemical reaction. This was accomplished by transferring 0.821 g 4,4’-bis(chloromethyl)-1,1’-biphenyl (Sigma-Aldrich) and 3.179 g (six equiv) of anhydrous iron(III) chloride (Sigma-Aldrich) as mediator into a 45 mL zirconium oxide milling vessel filled with 22 balls (10 mm, 3.19 ± 0.05 g) of the same material. Consequently, the vessel was transferred into a P7 premium line (Fritsch GmbH) and milled at 500 rpm for 35 minutes. After the synthesis the resulting polymers were washed with 200 mL of water and 100 mL of ethanol and dried at 80 °C for 12 hours.

For the LAG experiments the same quantities were used and 1 mL of the given liquid was added prior to the milling. The synthesis of the reference HCP from solution was carried out according to the procedure of Schute et al. [11]. Nitrogen physisorption measurements were performed at 77 K on an Autosorb-IQ-C-XR and Quadrasorb (Quantachrome Instruments). High purity gases were used for physisorption measurements (N_2_: 99.999%). Specific surface areas (SSA_BET_) were calculated using the equation from Brunauer, Emmet and Teller (BET) in a relative pressure range that fits to the consistency criteria proposed by Rouquerol and Llewellyn. Pore size distributions were calculated using the quenched solid density functional theory (QSDFT) method for carbon (slit pores, equilibrium kernel) on the adsorption branch. Total pore volumes were determined from the adsorption branch at *p*/*p*_0_ = 0.95. Prior to physisorption experiments, all samples were activated at 353 K for 24 h under vacuum. Ball mill syntheses were carried out in a Fritsch Pulverisette 7 premium line planetary ball mill. Infrared spectroscopy (IR) was carried out on a Bruker Vertex 70 with a Specac Golden Gate ATR unit. A resolution of 2 cm^−1^ was utilized and the resulting spectra were treated with ATR-correction by the OPUS 6.5 software. Powder X-ray diffraction (PXRD) patterns were collected in transmission geometry (MYTHEN 1K detector) with a STOE STADI P diffractometer operated at 40 kV and 30 mA with a Ge monochromator using Cu Kα_1_ radiation. Scanning electron microscopy (SEM/EDX) images were obtained using a Hitachi SU8020 SEM equipped with a secondary electron (SE) detector. Prior to the measurement the samples were prepared on an adhesive carbon pad and sputtered with gold to obtain the necessary electron conductivity. Thermogravimetric analysis (TGA) was performed on a Netzsch STA 409 PC/PG system using alumina crucibles under argon stream with the heating rate of 10 K min^−1^. The vapor sorption experiments were carried out using a Autosorb iQ from Quantachrome equipped with a vapor source and a heated manifold. Before evaporation the adsorptive solvent was dried using molecular sieves 4 Å and degassed under vacuum.

## Supporting Information

File 1 Additional information and figures.
