# Supplementary material for: Mechanochemical synthesis of hyper-crosslinked polymers: influences on their pore structure and adsorption behaviour for organic vapors
Source: Beilstein J Org Chem. 2019 May 24;15:1154–61. doi: 10.3762/bjoc.15.112 (PMC6541366; doi:10.3762/bjoc.15.112)
Supplement: File 1 — Additional information and figures. [file Beilstein_J_Org_Chem-15-1154-s001.pdf]

BEILSTEIN JOURNAL OF ORGANIC CHEMISTRY

Supporting Information

for

Mechanochemical synthesis of hyper-crosslinked polymers: influences on their pore structure and adsorption behaviour for organic vapors

Sven Grätz, Sebastian Zink, Hanna Kraffczyk, Marcus Rose and Lars Borchardt

Beilstein J. Org. Chem. **2019,** *15,* 1154–1161. doi:10.3762/bjoc.15.112

Additional information and figures

License and Terms: This is a supporting information file under the terms of the Creative Commons Attribution License (http://creativecommons.org/licenses/by/4.0). Please note that the reuse, redistribution and reproduction in particular requires that the authors and source are credited.
The license is subject to the *Beilstein Journal of Organic Chemistry* terms and conditions: (https://www.beilstein-journals.org/bjoc)

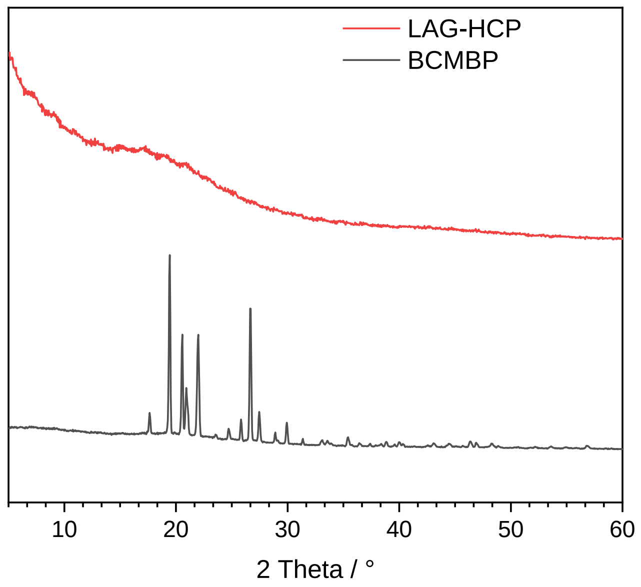

Figure S1: Powder XRD diffractograms of LAG-HCP and the monomer BCMBP. No contaminations in the armorphous polymer have been detected.

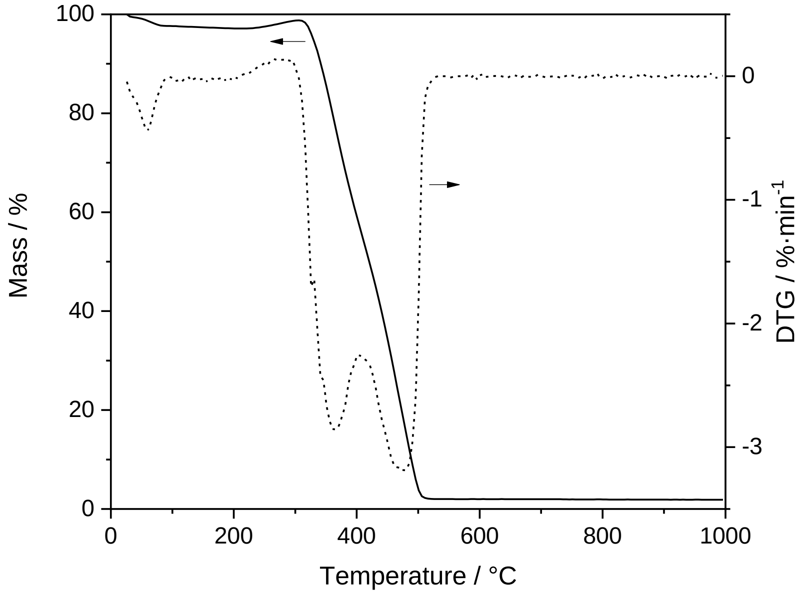

Figure S2 TGA curve of NG-HCP under air showing two major degradation steps with peaks at 365 °C and 475 °C. The almost complete degradation hints to only minor contaminations by the ball-milling process with oxidant or milling material.

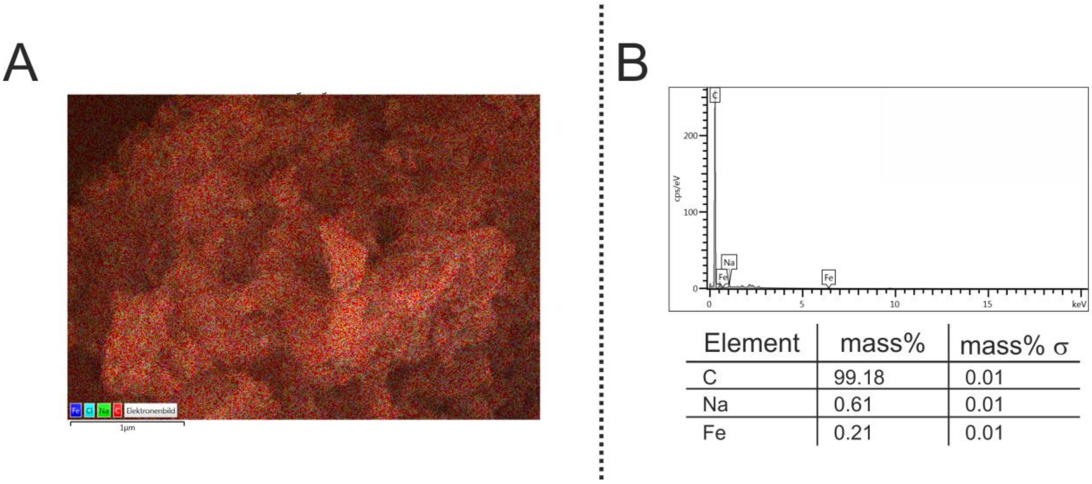

Element	mass%	mass% σ
C	99.18	0.01
Na	0.61	0.01
Fe	0.21	0.01

Figure S3: EDX data obtained via SEM. A: Overlay picture of the EDX scan. B: Measured EDX spectrum with the calculated mass % of the investigated elements. Cl was not found in the EDX scan and gold was used for sputtering and thus removed.

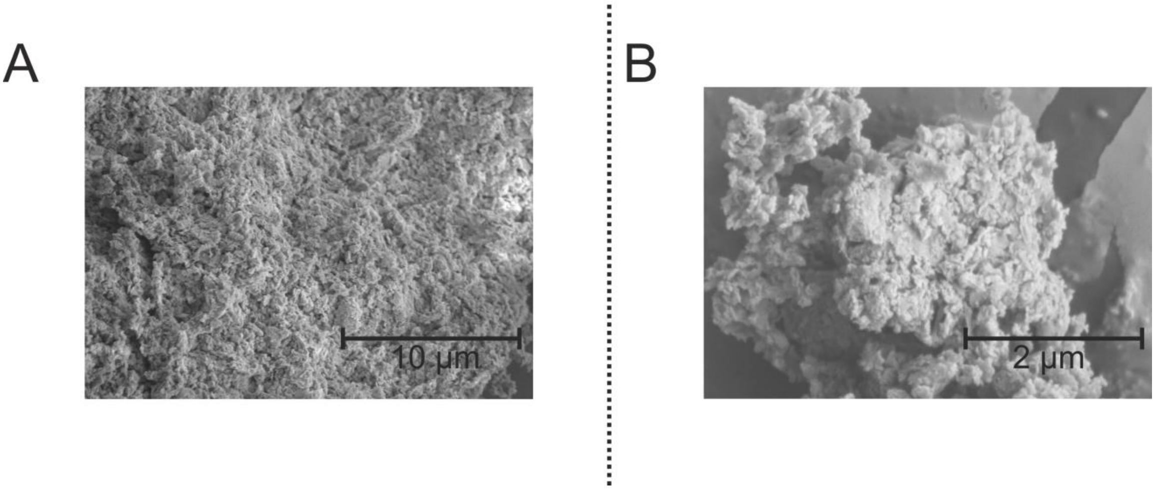

Figure S4: SEM micrographs of LAG-HCP at two different magnifications. We could not observe any differences between NG and LAG samples.

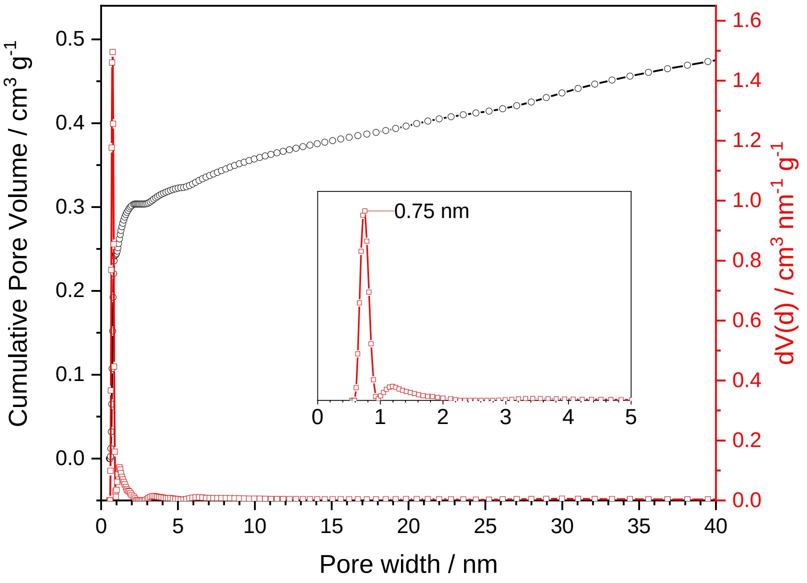

Figure S5: Pore size distribution of the NG-HCP from the nitrogen physisorption experiments via DFT calculations. (Calc. Model: N_2 at 77 K on carbon (slit/cylindr./sphere pores, QSDFT adsorption)

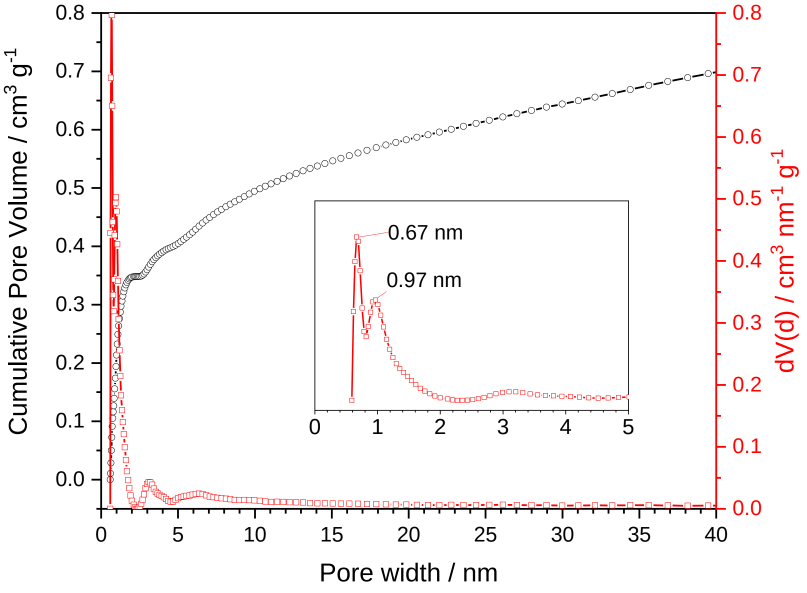

Figure S6: Pore size distribution of the LAG-HCP via from the nitrogen physisorption experiments DFT calculations. (Calc. Model: N_2 at 77 K on carbon (slit/cylindr./sphere pores, QSDFT adsorption)

DOE

Design of experiments describes a methodological variation of parameters in order to get the most information out of a minimum number of experiments. The data is therefore collected and a regression function is calculated in order to fit the investigated result (e.g., SSA) with the chosen parameters in order to determine their influence. Since it is a statistical approach the variance of the experiment is taken into consideration as well. This can be accomplished by repeating certain experiments. It is beneficial to repeat the centre points that are introduced into the set-up to detect non-linear behaviour because all their parameters are at under non-extreme conditions. The choice of parameters and their step size is crucial for precise results. It should be noted that it is in general better to choose them in such a matter as to obtain maximum effects, to better differentiate the effects from the normal variance. Furthermore the runs are conducted in a randomized order to spread influence from random disturbances (different batches of starting material, lab temperature, etc.) evenly over all experiments. For further details, please refer to books on this subject [1,2].

Table S1: DOE setup for the synthesis of NG-HCP

Entry	Milling time / min	RPM / min^{-1}	Equiv $FeCl_3$	SSA_{BET}[b] / m^2g^{-1}
1	35	500	6	874
2	10	200	2	7.5
3	10	200	10	38
4	60	800	2	812
5	60	200	10	67
6	35	500	6	908
7	60	200	2	13

8	10	800	2	820
9	60	800	10	630
10	10	800	10	792

The main effect plots for the obtained SSA of the HCP from the reaction are given in Figure S7. The main effect plots can give one an idea of the influence of a single parameter and helps to visualize the results from Table S1. The main influence is the rotational speed. While low speeds lead to an incomplete reaction with a non-porous material, too high speeds result in a partial thermal degradation of the polymer.

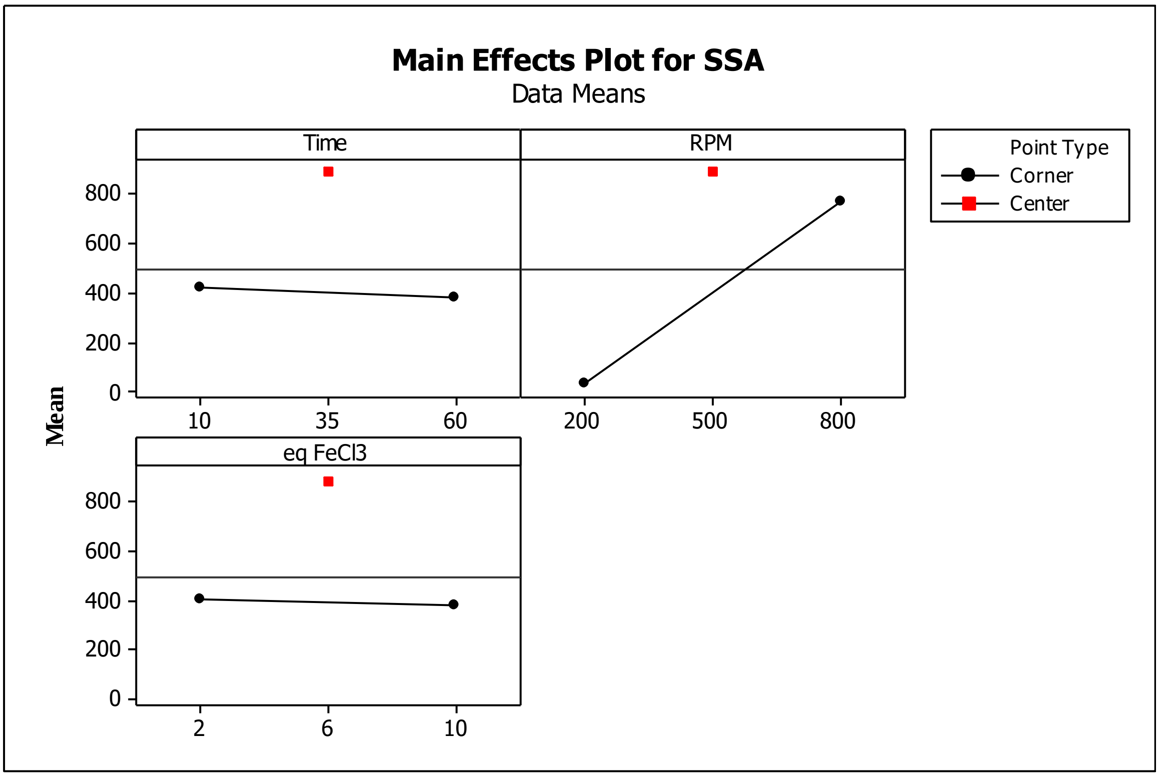

Figure S7: Main effect plot derived from the DOE data for the SSA showing the crucial impact of the rotational speed compared to the other two investigated parameters.

References

1. J. Antony, Design of experiments for engineers and scientists

2. K. Fang, J. Fan and G. Li, Contemporary multivariate analysis and design of experiments, World Scientific, 2005.
